# The First Line of Defense: The Effects of Alcohol on Post-Burn Intestinal Barrier, Immune Cells, and Microbiome

**DOI:** 10.35946/arcr.v37.2.06

**Published:** 2015

**Authors:** Adam M. Hammer, Niya L. Morris, Zachary M. Earley, Mashkoor A. Choudhry

**Affiliations:** Adam M. Hammer, Niya L. Morris, and Zachary M. Earley are graduate researchers in the Alcohol Research Program and the Burn and Shock Trauma Research Institute and doctoral candidates in the Program in Integrative Cellular Biology, Loyola University Chicago Health Sciences Division, Maywood, Illinois. Mashkoor A. Choudhry, Ph.D., is a professor in the Departments of Surgery and Microbiology and Immunology, Stritch School of Medicine; and a research investigator in the Alcohol Research Program and the Burn and Shock Trauma Research Institute, Loyola University Chicago Health Sciences Division, Maywood, Illinois.

**Keywords:** Alcohol use, abuse, and dependence, alcohol consumption, alcohol exposure, alcohol effects and consequences, burns, immunity, immune cells, microbiome, intestine, gut, intestinal lumen, intestinal barrier, bacteria, sepsis, organ failure, trauma, T cells, neutrophils, dysbiosis, human studies, animal models

## Abstract

Alcohol (ethanol) is one of the most globally abused substances, and is one of the leading causes of premature death in the world. As a result of its complexity and direct contact with ingested alcohol, the intestine represents the primary source from which alcohol-associated pathologies stem. The gut is the largest reservoir of bacteria in the body, and under healthy conditions, it maintains a barrier preventing bacteria from translocating out of the intestinal lumen. The intestinal barrier is compromised following alcohol exposure, which can lead to life-threatening systemic complications including sepsis and multiple organ failure. Furthermore, alcohol is a major confounding factor in pathology associated with trauma. Experimental data from both human and animal studies suggest that alcohol perturbs the intestinal barrier and its function, which is exacerbated by a “second hit” from traumatic injury. This article highlights the role of alcohol-mediated alterations of the intestinal epithelia and its defense against bacteria within the gut, and the impact of alcohol on intestinal immunity, specifically on T cells and neutrophils. Finally, it discusses how the gut microbiome both contributes to and protects the intestines from dysbiosis after alcohol exposure and trauma.

Each year 2.5 million people die from alcohol abuse and its related morbidities worldwide, making alcohol related deaths among the highest preventable causes of death, and the greatest cause of premature death and disability in men between ages 15 and 59 (World Health Organization 2011). Alcohol abuse predisposes individuals to life-threatening conditions such as alcoholic liver disease (ALD), acute respiratory distress syndrome (ARDS), sepsis, and multiple organ failure (MOF) (Bird and Kovacs 2008; Molina et al. 2003; Purohit et al. 2008). Further, studies show that intoxication often plays a role in physical injury (Pories et al. 1992). Data demonstrate that a majority of patients admitted to the hospital for traumatic injury have detectable blood alcohol levels at the time of admittance (Grobmyer et al. 1996; Jones et al. 1991; Maier 2001; McGill et al. 1995; McGwin et al. 2000; Silver et al. 2008). These patients generally require more extensive care than patients who have not been drinking. They more frequently require surgical intervention, experience higher susceptibility to infection, and have longer hospital stays (Silver et al. 2008). Supporting these observations, experimental data suggest that alcohol at the time of trauma results in more severe pathology in animal models (Choudhry and Chaudry 2008; Messingham et al. 2002; Molina et al. 2003, 2013). As a result, researchers estimate that in the United States alone, trauma and alcohol-related expenses to society total $185 billion annually (Li et al. 2004).

The disruptions to human biology that underlie the association between alcohol and these conditions bear exploring. The intestine, where alcohol first meets with digestive and immune mechanisms, is a primary source of alcohol-related pathologies. Here, alcohol and its metabolites encounter the physical barrier lining the gut that prevents invading pathogens from moving into the body. They also come into contact with a particularly complex frontier where the immune system must distinguish between commensal bacteria that normally colonize human intestines, and foreign microbes that cause disease. Any disruption of these systems by alcohol certainly could contribute to inflammatory states in the body that may in turn lead to serious conditions such as sepsis and MOF.

In support of these possibilities, data has shown that acute alcohol exposure negatively affects the function of the intestines, and this is exacerbated by a second traumatic insult such as burn injury (Akhtar et al. 2009, 2011; Li et al. 2008*a*, 2009*a*, 2011*a*, 2012; Rendon et al. 2012, 2013, 2014). The consequences of disruptions to the intestinal barrier, immune cells, and microbiome (see Glossary) can be observed within 24 hours following injury, and likely contribute to the life-threatening complications mentioned above. Thus, understanding how both acute and chronic alcohol exposure disrupt the homeostatic gastrointestinal tract is paramount. This article will review relevant studies examining the role of gut epithelia in defense against pathogenic bacteria within the gut and the impact of alcohol on intestinal immunity, highlighting T cells and neutrophils. Finally, it will review how the gut microbiome plays a role in maintenance of gut barrier integrity following alcohol exposure and trauma.

## Intestinal Anatomy and Histology

To fully understand the intricate relationships among the gut barrier, immune system, and microbiome, gastrointestinal (GI) anatomy requires review. The spatial relationships established between the lumen and barrier of the gut are essential for the proper function of the GI tract in digestion and nutrient absorption. The GI tract is a continuous tube that begins at the mouth and ends at the anus. The small and large intestines function mainly to absorb nutrients and water, and this review will focus on these organs.

**Figure 1:**
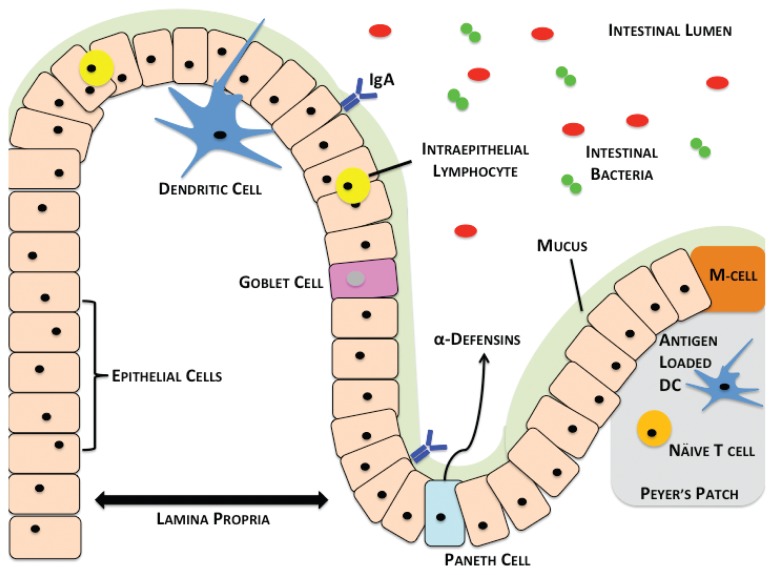
Overview of the intestinal barrier, immune cells, and microbiome. Lumenal bacteria (red and green) are relegated to the lumen of the intestine by the intestinal barrier composed of the mucus (green), which contains IgA bound antibodies (blue) and epithelial cells. The epithelial-cell layer contains intraepithelial lymphocytes (yellow) and mucin-secreting goblet cells (pink). At the base of the intestinal crypts lie Paneth cells (light blue), which secrete alpha-defensins. Directly below the epithelial layer lies the lamina propria. Dendritic cells sample the lumenal bacterial contents and migrate to Peyer’s patches (gray) within the small intestine, where they interact with T cells (orange). M cells allow the passage of antigens into Peyer’s patches for uptake by resident antigen presenting cells.

The small intestine is divided into three regions: the duodenum, jejunum, and ileum, respectively. At the distal end of the ileum lies the cecum, which connects the small and large intestines. From the cecum, the large intestine (colon) is composed of four regions: the ascending, transverse, descending and sigmoid colon, respectively, terminating in the rectum and anus. The small and large intestines are held in place to prevent twisting by the mesentery, which also contains the mesenteric lymph nodes (MLNs). As shown in figure 1, the small and large intestines at the histological level contain a barrier of mucous and epithelial cells that block the translocation of bacteria in the lumen to sites in the body beyond the intestines. Just below the intestinal epithelia lies a layer of loose connective tissue called the lamina propria (LP), which connects the surface mucosal epithelium to the basement muscularis mucosae. The LP also contains a large number of intestinal immune cells. In addition, specialized regions within the small intestine called Peyer’s patches (PPs) serve as lymphoid follicles, where naïve immune cells differentiate into a variety of mature immune cell subsets.

When a pathogen invades through the gut, the intestinal barrier and the immune cells in it mount a response to prevent infection. However, the picture gets more complex because of the gut microbiome, the mix of commensal bacterial species colonizing the lumen. The immediate proximity of the intestinal immune cells to the bacteria within the lumen presents a major challenge for homeostatic regulation. Thus, the interactions between the immune cells, intestinal barrier, and lumenal microbiome are of major interest in all areas related to pathology associated with the intestines. Alcohol modulates all of these components, and a disruption of any one can result in serious disease and/or infection that can affect all regions of the body.

## The Homeostatic Intestinal Physical Barrier

Looking more closely at the meeting point of the lumen with the intestinal wall, the intestinal physical barrier consists of a layer of mucus and epithelial cells that line the lumen and provide a crucial first line of defense against pathogens. Starting from the lumen, the first component of the physical barrier is a mucus layer. Mucus offers protection from the lumenal bacterial content and also lubricates the intestinal walls for passing bile (Bollinger et al. 2006; Groschwitz and Hogan 2009; Peterson and Artis 2014; Valatas and Kolios 2009). Immediately below the mucus layer, a single layer of epithelial cells forms a second barrier featuring tight junction protein complexes that adhere adjacent cells to each other (Peterson and Artis 2014; Ulluwishewa et al. 2011). The body maintains this barrier by regulating the proliferation and apoptosis of epithelial cells (Peterson and Artis 2014). Together, the mucus layer and epithelial cells of the intestinal barrier minimize interactions of inflammatory host immune cells with the lumenal bacteria.

### Mucus Layer

The mucus layer is a key component of the physical barrier and is formed by a glycoprotein, mucin (mainly mucin-2). Goblet cells found in the intestinal epithelial layer secrete mucin (Kim and Ho 2010). Mucin contains a glycosylated peptide backbone, which creates an incredibly viscous mucus layer effective at preventing pathogen penetration (Hartmann et al. 2013). Recently, a study found that the small intestine has a porous mucus layer that allows uptake of mucin-2 (MUC2) by intestinal dendritic cells (DCs) (see “Primer on the Immune System” in this issue). DCs containing MUC2 were able to generate anti-inflammatory responses through β-catenin and NFκB-mediated mechanisms, giving rise to a newly identified homeostatic role for the intestinal mucosa (Shan et al. 2013).

### Epithelial Layer

**Figure 2:**
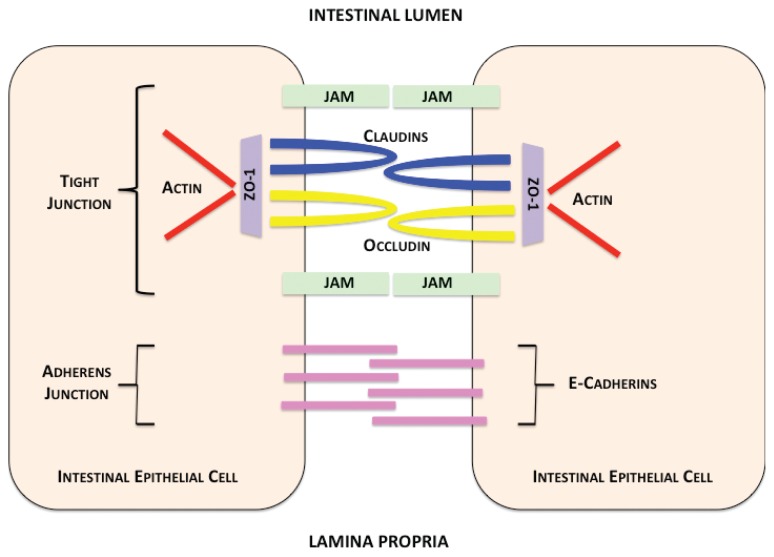
Intestinal epithelial-cell junctions. Contents within the intestinal lumen are prevented from passing between epithelial cells by apical tight-junction complexes. Tight junctions are composed of claudin proteins (blue) and regulated by occludin proteins (yellow). Claudin and occludin proteins are transmembrane proteins attached to an adaptor molecule, zonula occludins protein 1 (ZO-1) (purple), which anchors tight-junction proteins to intracellular actin (red). Alcohol causes disruption of occludin and ZO proteins by an unknown mechanism. Junctional adhesion molecules (JAMs) (green) also support tight-junction interactions. Intestinal epithelial cells are further supported by adherens molecules, including E-cadherins (pink), which also contribute to cell–cell contact. These junctions allow selective separation of the intestinal lumen (top) and lamina propria (bottom).

The mucus layer is not impenetrable, however, and the tight junction complexes between the epithelial cells below the mucus layer play a crucial role in providing a second level of protection. Tight junctions (figure 2) are multi-protein complexes consisting of transmembrane, scaffold, and adaptor proteins, which play an indispensable part in the maintenance of barrier function (Ivanov 2012). The proteins of tight junctions form a paracellular seal and function as a selectively permeable barrier between adjacent epithelial cells. They allow nutrients from food to pass out of the lumen while blocking passage of bacteria. Among the transmembrane proteins making up tight junctions are occludin, claudins, tricellulin, and junctional adhesions (Ulluwishewa et al. 2011). Although the function of occludin proteins is unknown, they are not essential for tight junction formation but appear instead to be instrumental in the regulation of the junctions (Balda and Matter 2008; Forster 2008; Groschwitz and Hogan 2009). Claudins are a family of both tissue- and cell-type–specific proteins considered to be the main structural components of the tight junctions. A third class of proteins found in tight junctions are junction-associated adhesion molecules (JAMs); however, little is known about their contribution to tight junction function and assembly (Balda and Matter 2008; Forster 2008; Groschwitz and Hogan 2009).

In addition to the transmembrane proteins that constitute the paracellular barrier, tight junctions also contain a complex system of adaptor molecules and scaffold proteins that mediate crosslinks between the transmembrane proteins and the actin cytoskeletons within epithelial cells. Besides forming tight junctions, intestinal epithelial cells themselves constitute a dynamic community of cells. The crypt-villus axis (see Glossary) allows constant regeneration of cells by differentiation and migration of cryptic stem cells to maintain barrier integrity. This balance of apoptosis and proliferation enables normal intestinal barrier function (Peterson and Artis 2014).

## Intestinal Physical Barrier Following Alcohol Exposure and Trauma

Disruptions in either the intestinal mucus or epithelial barrier can result in pathogenic bacterial translocation. This can lead to systemic infections, sepsis, and multiple organ failure, which underscores the importance of maintaining barrier integrity (Choudhry et al. 2000, 2004; Napolitano et al. 1995). Alcohol exposure can cause disruptions in all components of the intestinal barrier (Farhadi et al. 2003; Keshavarzian and Fields 2003). Such alterations may subsequently lead to an increase in bacterial translocation and infection among hospitalized trauma patients who have detectable blood alcohol levels at the time of their admittance (Bird and Kovacs 2008; Maier 2001; McGill et al. 1995; Molina et al. 2013; Silver et al. 2008; Valatas and Kolios 2009). Researchers have started to identify alcohol’s specific effects on different parts of the physical barrier.

As the first line of defense against pathogenic organisms within the intestinal lumen, the mucus layer and its alteration by alcohol exposure are of particular research interest. Grewal and Mahmood (2009) investigated the role of chronic alcohol exposure on mucin production in a rat model. They demonstrated that prolonged alcohol exposure (25 to 56 days) resulted in increased mucin production. This study also discovered that several components of the mucin biochemical composition were altered following prolonged alcohol exposure. Modulation of glycosylation and enzymatic activity within the mucus layer could potentially affect the barrier’s integrity, as these sites could begin to harbor adherent pathogenic bacteria (Van Klinken et al. 1995). In contrast to this finding, others have shown that chronic alcohol exposure results in decreased mucin production in the intestines of rats (Slomiany et al. 1997, 2000). Further-more, Hartmann and colleagues (2013) demonstrated that MUC2 knockout mice are less susceptible to bacterial overgrowth and translocation following chronic alcohol exposure and are thus less prone to alcoholic liver disease. These findings suggest a relationship between alcohol exposure and mucus production. Further investigation will be required to establish the effects of alcohol on mucin production and to elucidate the mechanism by which alcohol alters the intestinal mucus layer.

Not surprisingly, alcohol and trauma also disrupt the integrity of tight junction complexes between intestinal epithelial cells (Choudhry et al. 2002; Li et al. 2008*a*; Tang et al. 2009). An in vitro study showed that Caco-2 human intestinal epithelial cells exposed to a daily regime of alcohol demonstrated a reduction in membrane localization of the adherens protein ZO-1. Furthermore, allowing the alcohol-treated cells to “recover” from alcohol exposure by culturing them for 2 weeks in alcohol-free media improved ZO-1 localization (Wood et al. 2013). Studies by Rao and colleagues have also demonstrated that acetaldehyde, a metabolite of alcohol, results in similar disruption of occludin and ZO-1 proteins by altering their phosphorylation status (Atkinson and Rao 2001; Dunagan et al. 2012; Rao 2008). Another study conducted by Ma and colleagues (1999) using Caco-2 cells showed identical perturbation of ZO-1 proteins. The study further demonstrated that alcohol activates an enzyme, myosin light-chain kinase (MLCK), that phosphorylates myosin regulatory light-chain (MLC), promoting its interaction with actin to cause cytoskeletal sliding (Ma et al. 1999). This interaction is important in tight junction function and may be one cause of the alcohol-related disruption of tight junctions in intestinal epithelial cells (Groschwitz and Hogan 2009). Zahs and colleagues (2012) examined the role of MLCK in gut barrier disruption following combined binge alcohol exposure and burn injury. They showed that the combination of alcohol intoxication and burn injury results in both elevated MLCK and phosphorylated MLC and decreased co-localization of both occludin and ZO-1. Such changes could alter barrier permeability.

In an in vivo study of acute alcohol exposure and burn injury in rats, Li and colleagues (2012) showed that the combined insult resulted in a significant reduction in phosphorylation and expression of occludin and claudin-1, which was correlated with increased epithelial cell apoptosis. Yoseph and colleagues (2013) further demonstrated that the combination of chronic alcohol and cecal ligation and puncture (CLP)-sepsis resulted in elevated intestinal epithelial apoptosis as well as decreased proliferation of cells compared to CLP-sepsis alone. Clearly, exposure to alcohol and trauma greatly affects all components of the intestinal physical barrier through changes in mucosal production and biochemical structure, disruptions of tight junction protein complexes, and increasing susceptibility to apoptosis in epithelial cells. The mechanisms by which alcohol and trauma cause these alterations are just beginning to be elucidated. Future work will focus on how to prevent such disruptions.

## The Intestinal Immune System

**Figure 3:**
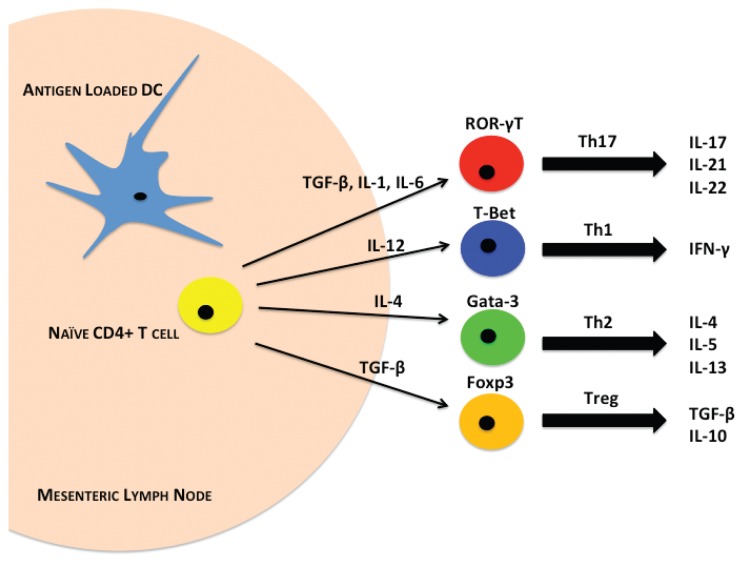
Intestinal CD4^+^ T-cell differentiation. Antigen-loaded dendritic cells (DCs) interact with naïve CD4^+^ T cells (yellow) in mesenteric lymph nodes through MHC-II molecules. DCs secrete different cytokines depending on this interaction. Following alcohol and burn injury, antigen-presenting cells (APCs) such as DCs may have a significantly altered cytokine expression profile. The cytokine profiles present lead to the expression of different transcription factors that promote differentiation of T cells into either Th17 (red), Th1 (blue), Th2 (green), or Treg (orange) phenotypes. These T-cell subsets secrete different cytokines that lead to inflammatory or immunosuppressive immune responses. Combined alcohol and burn injury has been shown to suppress T-cell cytokines including interferon (IFN)-γ, interleukin (IL)-17, and IL-22 from T cells.

Beyond the physical barrier, the next line of defense against invading pathogens is the immune system within the gut, which has the most difficult task in the body. Not only does it protect the host from invading pathogens, but it also maintains homeostasis with the vastly diverse microbiome within the intestinal lumen. The immune system must distinguish between commensal and pathogenic bacteria so that it does not mount a damaging autoimmune inflammatory response. The immune cells that carry out these tasks comprise parts of both innate and acquired immune functions. They can be found in all areas of the intestines, especially in regions called gut associated lymphoid tissue (GALT). GALT includes the gut epithelium, PPs, MLNs, and LP (Choudhry et al. 2004; Mowat and Viney 1997). Intestinal T cells are found in GALT sites and exist closely with antigen presenting cells (APCs), such as DCs and macrophages, that aid in T cell differentiation and activation (figure 3). Scientists are beginning to define the roles of macrophages and DCs in gut immune functions following alcohol exposure or trauma, as well as the initial innate immune responses that occur following these insults. These immune cells activate or suppress one another using highly complex chemical signaling pathways that researchers are beginning to uncover. Alcohol could produce disruptive effects at any point along these pathways (see figure 3).

### Innate Immunity

A key part of the innate immune response, neutrophils, or polymorphonuclear leukocytes (PMNs), make up a significant portion of the innate immune cells present in humans. They play integral roles in initial responses to infection including degranulation and phagocytosis (Amulic et al. 2012). It appears that one of the main functions of gut neutrophils under homeostatic conditions is to prevent the translocation of bacteria across the epithelial barrier (Choudhry et al. 2002; Kuhl et al. 2007; Li et al. 2008*b*). In addition, IL-17 cytokine released by activated T cells known as Th17 cells supports an inflammatory immune response through recruitment of neutrophils (Hundorfean et al. 2012). It is important to note that the role of neutrophils under pathologic conditions in the intestines remains unclear. In models of inflammatory bowel disease (IBD), different studies have shown neutrophils to be beneficial (Kuhl et al. 2007; Zhang et al. 2011), harmful (Kankuri et al. 2001; Natsui et al. 1997), or indifferent (Yamada et al. 1991). Interestingly, understanding of the function of neutrophils within the intestines of mice and humans has diverged slightly as studies show that murine neutrophils secrete defensins (see Glossary), whereas human neutrophils do not (Ganz 2003; Ouellette and Selsted 1996; Risso 2000).

### Neutrophil Activity Following Alcohol Exposure and Trauma

Following alcohol intoxication and trauma, neutrophil infiltration increases into different organs, including the lungs and intestines (Akhtar et al. 2009; Bird et al. 2010; Li et al. 2008*b*; Scalfani et al. 2007). Although the role of neutrophils is unclear in disease models such as IBD, neutrophils appear to have detrimental effects after alcohol exposure and trauma (Li et al. 2008*b*). Several studies have found that the inflammatory microenvironment following alcohol exposure and/or trauma may allow neutrophils to exacerbate tissue damage in numerous organs including intestine (Amin et al. 2007*a*,*b*; Bird and Kovacs 2008; Li et al. 2007, 2008*a*, 2011). Studies in animal models provide details surrounding neutrophil activity after alcohol intoxication and trauma. These publications show that not only are neutrophils recruited by the pro-inflammatory cytokines IL-6 and IL-18, but they also have a prolonged presence at the injury sites (Akhtar et al. 2009; Scalfani et al. 2007; Zahs et al. 2013). Scientists do not know whether IL-6 and/or IL-18 directly recruit neutrophils, or whether these cytokines signal through other molecules such as monocyte chemoattractant-1 (MCP-1) or myeloperoxidase (MPO) (Li et al. 2011; Rana et al. 2005). They also do not know what role alcohol plays in neutrophil recruitment. However, previous work showed that alcohol intoxication leads to increased recruitment of neutrophils to the intestine following ischemic injury (Tabata and Meyer 1995). One proposal suggests that this may occur through upregulation of intestinal ICAM-1 expression following ischemic/reperfusion injury (Olanders et al. 2002). Once at the injury site, neutrophils secrete superoxide anions that kill any invading pathogens entering through the compromised intestinal barrier (Li et al. 2008*b*, 2011). Although this response is helpful at initially protecting from invading pathogens, prolonged neutrophil responses mediate tissue damage in multiple organs under inflammatory conditions (Fukushima et al. 1995; Partrick et al. 2000). Further studies will be necessary to determine how neutrophils respond following alcohol exposure, and also how they mediate the subsequent adaptive immune response.

### Adaptive Immunity

T lymphocytes form a large part of the adaptive immune response in the intestine. Under homeostatic conditions, the balance between inflammatory and immunosuppressive T cells is maintained through cell-to-cell cytokine signaling. Although the intestines contain a large and diverse population of T lymphocytes, the major subsets of resident T cells within the gut include Th1, Th2, Th17, and T-regulatory (Treg) cells (Belkaid et al. 2013). The default T cell response in the intestines under normal conditions is immunosuppressive. This occurs through the production of TGF-β, primarily by APCs, which drives Treg development (figure 3). In addition to TGF-β, IL-4 production drives Th2 cell development and B cell IgA antibody production. IgA also maintains gut homeostasis, in part by regulating the microbiome (Weaver et al. 2006).

The production of these immunomodulatory cytokines largely depends on resident DCs that sample the lumenal contents at the epithelial barrier (Cerovic et al. 2014). DCs decipher commensal and pathogenic bacterial antigens to modulate appropriate T-cell development by a mechanism now under investigation (Cerovic et al. 2014). Naïve CD4^+^/Foxp3-T cells within GALT are driven toward specific T-cell phenotypes, depending upon the milieu of extrinsic factors present. Once activated, these T cells release cytokines to generate an immune response. Development of the Th1 phenotype depends on cytokines including IL-12, which is augmented by the presence of IL-18. IL-12 binds to its cognate receptor (IL-12R), which results in downstream signaling through the transcription factors STAT4 and T-box protein 21 (T-bet) (Amsen et al. 2009). Interestingly, recent reports show that STAT4 and T-bet may act in unison to drive Th1 differentiation. Thieu and colleagues (2008) have described a role for STAT4 in chromatin remodeling that promotes *Ifng* gene transcription by T-bet to drive Th1 differentiation. This signaling is initiated following antigen recognition on MHC-II molecules, whereupon Th1 cells secrete the cytokines IFN-γ and lymphotoxin alpha (LT-α), a member of the pro-inflammatory TNF family (Weaver et al. 2006). Some have hypothesized that Th1 cells may play a role in regulating innate mucosal responses; however, further investigation must confirm this (Belkaid et al. 2013). As mentioned above, other cytokines such as TGF-β keep development of Th1 cells in check under homeostatic conditions. TGF-β plays an important role in preventing the differentiation of naïve T cells into inflammatory phenotypes (Sansonetti and Di Santo 2007).

Th17 cells form the other major inflammatory T cell subset found in the intestines. Intestinal Th17 development also depends heavily on the cytokine milieu. It is largely driven by the presence of IL-6. More recent studies have implicated IL-23 in Th17 differentiation, but it appears that IL-23 may only augment Th17 differentiation as opposed to being an essential component (Maynard and Weaver 2009). IL-6 and IL-23, which are mainly produced by DCs and macrophages, signal through their cognate receptors on naïve CD4^+^ T cells, which in turn signal through the ROR-γT transcription factor. ROR-γT transcription drives Th17 cells to produce a host of different cytokines including IL-17A, IL-17F, IL-21, and IL-22 (Maloy and Kullberg 2008). Many contrasting studies have been published regarding the roles of Th17 cytokines. Although IL-17A and IL-17F are generally present under inflammatory conditions (Ahern et al. 2010; Leppkes et al. 2009; Wu et al. 2009; Yang et al. 2008), scientists have also observed contradictory protective roles of IL-17A in models of IBD (Yang et al. 2008). Fewer studies have examined the actions of IL-21 and IL-22, but both cytokines seem to play a protective role in epithelia regeneration following injury (Maloy and Kullberg 2008; Sonnenberg et al. 2010). Although it is clear that Th17 cells play an essential part in modulating intestinal inflammatory immune responses, more studies will be needed to elucidate their specific functions in homeostatic and diseased conditions within the intestines.

Balancing the inflammatory T cells within the intestines, modulatory T cells are an important subset made up of Th2 and Treg cells. Antigen-loaded DCs that have sampled the lumenal contents release IL-4 to drive the differentiation of Th2 cells. Activation of the IL-4 receptor leads to downstream signaling through the transcription factor STAT6, which mediates the expression of another transcription factor, Gata3 (Ansel et al. 2006). Gata3 plays a major role in mediating production of key Th2 cytokines IL-4, IL-5, and IL-13. Gata3 also prevents Th1 differentiation through its inhibitory effects on IL-12 receptor and STAT4 signaling (Amsen et al. 2009; Ansel et al. 2006). One of the most important roles for Th2 cells in the maintenance of gut homeostasis is their interaction with B cells to aid in the development of IgA-producing plasma cells. IgA antibodies function to regulate homeostasis of the microbiome, as well as act as a first line of immune defense against pathogens in the GI lumen. They are by far the most highly expressed class of antibodies in the intestines of humans (Mantis et al. 2011).

Treg cells also serve a critical function in modulating the immune responses within the intestines. Populations of Tregs within the gut derive both from thymic CD4^+^CD25^+^Foxp3^+^ precursors that migrate to the gut, as well as from the gut itself, where resident naïve CD4^+^ T cells are preferentially driven towards a Treg phenotype by TGF-β, IL-10, and Foxp3 expression (Fontenot et al. 2005). Studies show that the recognition of self-antigens presented by DCs initiates Treg activation (Hsieh et al. 2006; Nishikawa et al. 2005; Watanabe et al. 2005). After sampling the lumenal contents in the intestine, DCs migrate to MLNs where some present self-antigens on MHC-II molecules to naïve CD4^+^ T cells. Activation of T-cell receptors by self-antigens stimulates Foxp3 signaling to drive anti-inflammatory TGF-β and IL-10 secretion. In this regard, Tregs are able not only to inhibit inappropriate inflammatory responses to these self-antigens by Th1 and Th17 cells, but also to drive Th2 and subsequent IgA production to maintain intestinal homeostasis. More recent observations have demonstrated that T cell lineages can interconvert, specifically Treg-to-Th17 and Th17-to-Th1 (Lee et al. 2009; Zhou et al. 2009). In light of these studies, it is important to highlight that while each subset of T cells found in the intestines plays a crucial role in balancing homeostasis, these relationships are dynamic and can be altered by changes within the intestinal environment, such as those following alcohol exposure.

### Intestinal T Cells Following Alcohol Exposure and Trauma

Surprisingly, few studies in the current literature have examined the effects of alcohol specifically on intestinal immunity. However, alcohol has significant, well-documented impacts on immune cells at sites outside the intestine, including in the spleen, thymus, and on circulating lymphocytes (Curtis et al. 2013; Ippolito et al. 2013; Messingham et al. 2002). Intestinal studies suggest that alcohol may have inflammatory effects, and subsequently compromise the intestine’s ability to prevent bacteria from passing into the body.

Of course, an important consideration in studying the effects of alcohol on immune function is the nature of the alcohol exposure (acute vs. chronic). The authors examined the effects of alcohol exposure in an acute model, which is followed by a second traumatic burn injury. In this model, mice are given a single dose of alcohol to produce a blood alcohol level of 90–100 mg/dL 4 hours after alcohol administration, at which time they are given a full thickness ~12.5% total body surface area dorsal scald burn. Findings demonstrate that alcohol intoxication or burn injury alone does not cause significant changes to immune profiles within the gut in the first 24 hours. However, combined alcohol and burn injury lead to great perturbations resulting in high levels of inflammation accompanied by neutrophil infiltration, T-cell suppression, and bacterial translocation (Brubaker et al. 2013; Li et al. 2008*a*,*b*, 2011*a,b*, 2012*a,b*, 2013; Rendon et al. 2012, 2013, 2014; Zahs et al. 2013). These results clearly demonstrate that alcohol intoxication leads to greater susceptibility to secondary insults by sensitizing the immune system through an unknown mechanism.

Studies from the authors’ laboratory also show a decrease in Th1 cells, particularly in MLNs, paired with decreases in IL-12 following alcohol intoxication and burn injury (Choudhry et al. 2002; Li et al. 2006). Intriguingly, restoration of IL-12 following alcohol and burn treatment restores Th1 profiles of the cytokines IFN-γ and IL-2 via an ERK-dependent pathway (Li et al. 2009). IL-12 is largely produced by resident APCs, and thus alcohol intoxication and burn injury may have both direct (i.e., on T cells) and indirect (on APCs) effects on Th1 function. Diminished Th1 effector cells present following alcohol intoxication and burn injury may allow bacteria and other pathogens to progress across the intestinal barrier. However, future studies will further address the signaling pathway(s) involved.

The authors also examined the effect of alcohol and traumatic burn injury on intestinal Th17 cells. They previously discovered a decrease in IL-23 and the Th17 effector cytokines IL-17 and IL-22 in PPs following alcohol and burn (Rendon et al. 2014). Due to the decreased presence of IL-23, they examined the effects of adding IL-23 following alcohol and burn injury (Rendon et al. 2014). Interestingly, IL-23 restored IL-22 production in an aryl hydrocarbon receptor (AhR)–dependent fashion, but IL-23 had no effect on IL-17 levels. These data give new insight into the role of IL-23 in mediating Th17 IL-22 responses, but not IL-17 responses. Like Th1 cells, the suppression of Th17 cells in the context of the alcohol/burn model may mean enhanced susceptibility to bacterial translocation and infection. Future studies will further examine the role of both Th1 and Th17 cells and their functions following alcohol intoxication and trauma. Th2 and Treg activity following alcohol and burn injury also has not been well studied.

Another research group published recent studies examining the effects of alcohol on intestinal immunity in the context of chronic alcohol exposure followed by sepsis (Yoseph et al. 2013). Studies performed in this model showed disruptions in intestinal permeability similar to those in the studies discussed above. In addition, a significant increase in CD4^+^ production of IFN-γ and TNF-α was observed in alcohol-treated mice compared with controls (Yoseph et al. 2013). Interestingly, studies of non-alcoholic human sepsis patients have shown lower levels of IFN-γ and TNF-α production in the spleen, which highlights the fact that local and systemic immune responses may differ greatly regardless of the presence of alcohol (Boomer et al. 2011).

Only a few studies in the literature have examined the effects of alcohol alone on intestinal immunity (Sibley and Jerrells 2000). An early study by Lopez and colleagues (1997) examined the effects of both acute and chronic alcohol exposure on PPs. They observed a significant decrease in the total number of cells within PPs of mice given a brief alcohol exposure of 5 weeks. In a more chronic exposure model, mice receiving alcohol for 19 weeks showed both a significant decrease in total PP cells, as well as a significant reduction of T and B cells present in PPs (Lopez et al. 1997). This study was important in demonstrating that alcohol administration affects the mucosal immune system, particularly PPs, suggesting that alcohol may thus affect T-cell differentiation within the intestines.

**Glossary**

AhR: Aryl Hydrocarbon Receptor: Transcription factor that drives Th17 cell differentiation
β-Catenin: Transcription factor involved heavily in cell adhesion regulation
CD(4/8): Cluster of differentiation: proteins expressed on the surface of cells used to identify specific cell phenotypes
Crypt-Villus Axis: The plane that exists from the base of intestinal crypts to the tops of the villi. Epithelial cells divide from stem cells at the base of crypts and migrate to the tops of villi as they mature
C-Type Lectins: Carbohydrate binding proteins with a diverse range of functions, including mounting immune responses against pathogens
Defensins: Small proteins secreted by paneth cells that mediate defense against harmful microbes
Dysbiosis: Any perturbation in the normal intestinal microbiota
Extracellular Signal–Related Kinase (ERK): Signaling molecules that transmit a variety of intracellular signaling following activation
Foxp3: Transcription factor that drives regulatory T cell differentiation
Gata3: Trans-acting T-cell-specific transcription factor involved in the development of Th2 cells
Glycosylation: A post-translational modification that involves the attachment of a carbohydrate to the specific region of a protein to enhance its function
Intracellular Adhesion Molecule-1 (ICAM-1): Expressed mainly on endothelial cells and immune cells to mediate migration from circulation into tissues
Microbiome: The entire makeup of bacteria that inhabit the intestines
Nuclear Factor Kappa–Light-Chain Enhancer of Activated B Cells (NFκB): Transcription factor considered to be the master regulator of inflammation
Retinoic Acid-Related Orphan Receptor Gamma T (ROR-γT): Transcription factor that mediates Th17 development
Sepsis: Life-threatening whole-body inflammatory response in order to fight systemic infection
Signal Transducer and Activator of Transcription (STAT): Following receptor activation, STAT family proteins mediate transcription events to drive specific gene expression
T-Box Transcription Factor (T-bet): Transcription factor the mediates development of Th1 T cells
Zonula Occludins Protein 1 (ZO-1): Adherens trans-membrane junction proteins linking to the actin cytoskeleton to occludin and claudin proteins support tight junctions

A more recent study demonstrated that alcohol exposure causes disruption of the epithelial barrier in the stomach and upper intestines (Bode and Bode 2003). It has been reported than even a single dose of alcohol at binge consumption levels can result in epithelial barrier disruptions within the gut (Bode and Bode 2005). Interestingly, in an acute model of alcohol exposure, mice displayed higher numbers of Treg cells in the LP in response to barrier disruption (Boirivant et al. 2008). These results contrast with studies of chronic alcohol exposure that show increased levels of inflammatory neutrophil, Th1, and Th17 activation and production of IL-17A, IFN-γ, IL-1, and TNF-α (Bode and Bode 2005; Koivisto et al. 2008) Thus, acute alcohol exposure may result in suppression of inflammation, allowing pathogens past the intestinal barrier, while chronic exposure may produce an inflammatory state. In addition, one report with human subjects showed increases in IgA antibody production coupled with increases in TNF-α and IL-8 production in chronic alcoholics (Koivisto et al. 2008). Chronic alcohol consumption studies have reported significant effects on the liver and connected the inflammatory conditions observed in the intestines with alcoholic liver disease (Bode and Bode 2005; Koivisto et al. 2008).

## Microbiota and Intestine Immune Homeostasis Following Alcohol and Burn Injury

The adaptive T-cell response provides a critical component of pathogen protection, and innate responses conducted mainly by neutrophils also play a large role in maintaining intestinal homeostasis. Importantly, however, both of these immune responses are shaped by their interactions with the intestinal microbiome. The intestinal immune system encounters more antigens than any other part of the body. Therefore, the recognition of “self” and “non-self” antigens is critical to discriminate the harmless commensal microbiota and food antigens from harmful pathogenic microbes. In part, this equilibrium is established by the balance of effector T cells discussed earlier. Antigens from the intestinal microbiota presented in GALT by APCs shapes this balance of Treg/Th17 cells, which drives pro- or anti-inflammatory signaling.

In addition to affecting the T-cell balance, the composition of the intestinal microbiota facilitates development of lymphoid organs and directs immune cell responses and production of effector cytokines. Studies using germ free mice—that is, mice devoid of any microbes—reveal that these mice are more susceptible to colonization by pathogenic microbes; have small and undeveloped lymphoid organs; and show reductions in CD4^+^ and CD8^+^ T-cells, IgA secretion, and production of antimicrobial peptides (AMPs) including β-defensins and C-type lectins such as Reg3γ (Bouskra et al. 2008; Cash et al. 2006; Zachar and Savage 1979). Further, following combined alcohol and burn injury, Reg3β and Reg3γ are significantly decreased in the small intestines of wild-type mice (Rendon et al. 2013). Together, these findings suggest that following alcohol intoxication and injury, bacterial overgrowth and translocation may be partially mediated through the inhibition of AMPs.

Several recent studies demonstrate that certain bacterial species have specific effects on immune system balance. The commensal microbes, it turns out, are essential for regulating immune physiology and the innate and adaptive immune systems. One commensal, *Bacteroides fragilis,* produces an immunomodulatory molecule called polysaccharide A (PSA), which regulates the Th1 and Th2 balance and directs Treg development to protect against intestinal inflammation (Mazmanian et al. 2005; Round and Mazmanian 2010; Round et al. 2011; Xu et al. 2003). Mazmanian and colleagues (2005) showed that therapeutic treatment with PSA led to the production of anti-inflammatory IL-10 and alleviated intestinal inflammation in various models of IBD. Segmented filamentous bacteria (SFB), a group of Gram-positive bacteria, attach to small intestine epithelial cells and lead to the production of serum amyloid A (SAA). SAA then stimulates dendritic cells in the LP to secrete IL-6 and IL-23, which promotes Th17 cell differentiation and maturation (Ivanov et al. 2009). Littman’s laboratory and coauthor Ivanov and their team showed that germ-free mice have reductions of Th17 cells in the small intestine, but that levels could be restored by colonizing mice with feces taken from germ-free, SFB mono-colonized mice (Ivanov et al. 2009). Furthermore, they determined the specific membrane bound antigenic proteins of SFB that direct Th17 production (Yang et al. 2014). This bacterial group is also necessary for the secretion of IgA (Wu et al. 2011). Nevertheless, overgrowth of this bacterium may upset the Th17/Treg balance in favor of overactive Th17 cells. This shift can potentially lead to autoimmune diseases: inflammatory bowel disease, arthritis, and multiple sclerosis (Lee et al. 2011; Wu et al. 2010).

## The Intestinal Microbiota Following Alcohol Exposure and Trauma

Unexpectedly, few studies in the current literature have examined the effects of alcohol exposure on the microbiome within the intestines. A recent study examining the effects of chronic daily alcohol consumption found dysbiosis—a microbial imbalance—in the colons of rats after 10 weeks (Mutlu et al. 2009). Others have correlated microbial dysbiosis to alcoholic liver disease and demonstrated that administration of probiotics reduces hepatic inflammation associated with it (Mutlu et al. 2009; Wang et al. 2013). The work done by the authors showed that combined alcohol intoxication followed by traumatic burn injury results in a significant increase in bacterial translocation across the intestinal barrier (Choudhry et al. 2002; Kavanaugh et al. 2005; Li et al. 2012; Rendon et al. 2013), and this work is supported by a previous study (Napolitano et al. 1995). However, the long-term impact of alcohol on different microbiota and the host’s health and immune function remains to be shown. Classification of the healthy intestinal microbiome is clinically necessary for determining how alcohol may alter the microbiota composition and lead to disease development and progression. Thus, whether bacterial translocation after alcohol and trauma is related to changes in the microbiome remains largely unknown. Furthermore, studies are needed to establish whether changes in the biome have any role in epithelial barrier disruption following alcohol and burn injury.

## Future Directions and Perspectives

Taken together, the range of effects alcohol has on the intestines is extremely broad and alters all levels of intestinal homeostatic regulation. In parallel, alcohol exposure predisposes its users to more complications following major injury and trauma; however, the underlying mechanisms remain largely unexplored. Although studies have demonstrated that alcohol modulates the various components of the intestinal barrier, making any causal connections between these effects and complication from trauma requires more study. The balance of inflammatory and immunosuppressive T cells can be skewed following alcohol exposure. Current research suggests inflammatory conditions are mediated through both neutrophil infiltration and Th17 recruitment leading to tissue damage within the intestines. Whether alcohol influences this also needs to be explored. Many studies now show roles for the intestinal microbiome in developing the immune profiles within the intestines. Although few studies have explored whether alcohol exposure alters the composition of the microbiome, it is not far-fetched to hypothesize that this is likely the case. Together, the authors believe that the largest gap in the field remains the lack of mechanistic support for the changes observed following alcohol exposure with and without burn trauma. More studies are needed to understand the molecular signaling pathways mediating changes in the barrier, immune system, and biome to give a clearer understanding of the relationship between these components and how they overlap after alcohol and burn injury.
